# Sleep Patterns during the COVID-19 Lockdown in Spain

**DOI:** 10.3390/ijerph20064841

**Published:** 2023-03-09

**Authors:** Noelia Ruiz-Herrera, Amparo Díaz-Román, Alejandro Guillén-Riquelme, Raúl Quevedo-Blasco

**Affiliations:** 1 Faculty of Health Sciences, International University of La Rioja, 26006 Logroño, Spain; 2 Faculty of Psychology, University of Granada, 18011 Granada, Spain; 3 Faculty of Health Sciences, Valencian International University, 46002 Valencia, Spain; 4 Centre for Mind, Brain, and Behaviour Research (CIMCYC) and Faculty of Psychology, University of Granada, 18011 Granada, Spain

**Keywords:** coronavirus, pandemic, sleep, circadian rhythm

## Abstract

Background: To mitigate the spread of the coronavirus disease (COVID-19) pandemic, governments around the world adopted exceptional lockdown measures. This led to the disruption of normal life routines, including sleep. The aim of this study was to analyze differences in sleep patterns and subjective variables of sleep quality before and during lockdown. Methods: A sample of 1673 Spanish adults (30% men; 82% of the total were between 21 and 50 years of age) was evaluated. The following sleep variables were evaluated: Sleep latency, sleep time, number and duration of awakenings, sleep satisfaction, daytime sleepiness, and the manifestation of symptoms related to sleep problems. Results: Although 45% of people changed their sleep schedules (resulting in 42% sleeping longer during lockdown), sleep quality (37.6% worse), daytime sleepiness (28% worse), number of awakenings (36.9% more), and duration of awakenings (45% longer) were markedly worse. Statistical analyses indicated significant differences in all the evaluated sleep variables before and during lockdown in both men and women. Women reported less sleep satisfaction, and more symptoms related to sleep problems than men. Conclusions: A deterioration in the sleep patterns of the Spanish population, especially women, because of the lockdown declared due to the COVID-19 pandemic.

## 1. Introduction

In March 2020, the World Health Organization [1] declared the public health emergency an international pandemic due to the worldwide spread of the “coronavirus disease” (COVID-19). Consequently, the Government of Spain [2], like that of most countries in the world, was forced to decree certain actions and containment measures to prevent the spread of the virus to ensure people’s health and safety. A first state of alarm was declared, during which the health authorities and different public bodies established a series of measures aimed at trying to control and stop new infections and possible outbreaks. These measures were part of a new stage of lockdown for the Spanish population during the coming months and during the second state of alarm, in force until 9 May 2021.

The COVID-19 lockdown has entailed an important demand for the adaptive capacities of citizens in the face of a very novel stressful situation, generating a propitious scene for the emergence of psychological and physiological problems [3,4,5,6]. This was not only among the population forced to be at home but also among healthcare personnel [7]. The results of the study conducted on the health consequences derived from this situation show that a high percentage of the population suffered a moderate or serious increase in symptoms and behaviors of stress, anxiety, depression, post-traumatic stress disorder, psychological discomfort, and insomnia [5,6,8,9,10,11,12,13]. Concerning sleep, there is scientific literature assessing the effect of lockdown during COVID-19.

At the international level, Trakada et al. [14] conducted a study with 1908 participants from different countries in which they used a questionnaire with 13 items, six of which collected information on sleep. The authors showed that, although the sleep duration increased during a week of lockdown, 15% of participants described their sleep as bad and 37.9% described it as of medium quality. Furthermore, one third of the participants reported worse sleep quality during a week of lockdown than in normal conditions. Another Italian study, in which 1310 participants (students and workers) completed the Italian version of the Pittsburgh Sleep Quality Index (PSQI) [15], revealed that their sleep schedule had notably changed, as people went to bed and got up later and spent more time in bed. However, paradoxically, those participants also reported worse sleep quality [16]. These results are similar to those afterward reported by Marelli et al. [17] in their study with 400 participants (students and staff workers), in which they administered the Italian versions of the PSQI [15], the Insomnia Severity Index-ISI [18], and the reduced Morningness–Eveningness Questionnaire [19]. The authors noted a later bedtime, a longer sleep latency and time to wake-up, and worsening of sleep quality and insomnia symptoms, when compared before and during the COVID-19 emergency. Kocevska et al. [20] assessed 667 individuals in self-isolation using the ISI [21] and found that one fourth of the people with insomnia before the pandemic paradoxically experienced a significant improvement in their sleep quality. In contrast, the 20% of good sleepers before the pandemic experienced worse sleep during the lockdown measures. They also observed that the changes in sleep quality caused by the pandemic were associated with negative emotions and worry [21].

In Spain, in a large study conducted during the initial stage of lockdown, the Depression Anxiety Stress Scale-DASS-21 [22] was administered to more than 15,000 participants, along with the Impact of Event Scale (two items of this one were used to assess sleep characteristics [23]). The results of this study showed high rates of difficulties in initiating or maintaining sleep (23.9%), associated with a higher age, female sex, lower income, elderly care, alcohol consumption, depression, anxiety, and stress [24]. Another study with a sample size of 5220 participants found that women assessed with the PSQI had worse sleep quality [25]. This questionnaire and the Spanish version of the Satisfaction, Alert, Time, Efficiency, and Sleep Duration Questionnaire-SATED [26], and a modified version of the Epworth Somnolence Scale-ESS [27], were used by Targa et al. [28] in a sample of 71 participants. They observed a deterioration in sleep quality before and during the COVID-19 outbreak in parallel with an increase in negative mood.

On the basis of the presented data, it is possible to conclude that sleep and biological rhythms have been altered due to the opportunities to extend sleep time in the morning and take naps during the day [29]. In addition, it is necessary to take into account the environmental or situational variables that make it difficult to get to sleep [30,31,32] and were present during COVID-19 lockdown. Specifically, these are the deprivation of normal exposure to natural light, the breakdown of schedules and usual routines at different levels (work, social, family, and even leisure), together with the feeling of fear and uncertainty in the face of the disease, and the overall situation. Despite this, Spanish studies do not have large sample sizes in which pre- and during lockdown data are compared by means of both objective measures and subjective daytime sleepiness, fatigue, and sleep quality.

The aim of this study was three-fold: (a) to analyze if the lockdown established in Spain during the COVID-19 pandemic implied significant changes in sleep patterns; (b) to determine if potential disturbances in sleep patterns were associated with sleep quality and efficiency; and (c) to assess differences in sleep patterns and perceived sleep quality depending on sex.

## 2. Materials and Methods

### 2.1. Design and Participants

This is a cross-sectional observational study with a single evaluation involving a retrospective self-assessment. The total sample consisted of 1673 participants (69.34% women) from various regions of Spain. Participants had to fulfill the following inclusion criteria: be older than 18 years old, reside in Spain, sign the informed consent form and not report abnormal response patterns. Of the recruited sample, 13.15% were healthcare workers, 32.16% had children, 31.68% had people to care for, and 18.11% were suffering from some illness at the time of their participation in the study. The age distribution for men was as follows: 32 with 18–20 years (6.24% of the total men and 1.91% of the total sample), 218 with 21–35 years (42.5% and 13.03% of the total), 174 with 36–50 years (33.92% and 10.4% of the total), 75 with 51–65 years (14.62% and 4.48% of the total), and 14 with more than 65 years (2.73% and 0.84% of the total). In the case of women, the age distribution was as follows: 97 with 18–20 years (8.36% of the total women and 5.8% of the total sample), 599 with 21–35 years (51.64% and 35.8% of the total), 304 with 36–50 years (26.21% and 18.17% of the total), 147 with 51–65 years (12.67% and 8.79% of the total), and 13 with more than 65 years (1.12% and 0.78% of the total).

### 2.2. Instruments

An ad hoc questionnaire was used to assess the variables of interest. When possible, a categorical response scale was used to make online questionnaire completion easier and more comfortable, resulting in more complete responses and fewer doubts and errors. As a result, the developed questionnaire was as standardized as possible, and the following areas were assessed: (a) sociodemographic: questions regarding sex, age, academic level, if the profession was related to the health field, marital status, number of children, people in charge, and if the person evaluated had any serious illness at the time of the evaluation; (b) data related to sleep patterns: questions about bedtimes, wake-up times, naps, and the number and duration of awakenings; and (c) data related to perceived sleep quality: items of perceived sleep quality, daytime sleepiness, and tiredness.

### 2.3. Procedure

After the design of the questionnaire and the approval of the full project by the Ethics Committee of the University of Granada (see Institutional Review Board Statement), the *LimeSurvey* platform was used to computerize informed consent and the questionnaire. Participants were recruited through social media and snowball sampling. Sampling was conducted during one week in April 2020. Appropriate action was taken to control biases and preventing duplicate responses. The database (with anonymous data) was downloaded from platform as a CSV format, and files were imported into the statistical software. Participants who completed less than 80% of the questionnaire were excluded from the analyses. Participants’ responses were also reviewed to detect abnormal response patterns, and no extreme or illogical responses were observed in any case.

### 2.4. Data Analysis

The graphic representation of data through alluvial plots was the analysis procedure chosen to facilitate the interpretation of the data due to the quantity of changes detected pre- and during-COVID-19 lockdown and the large number of variables analyzed. The Chi square test was also used to confirm if the results were significant. Each variable was separately assessed by comparing pre- and during -lockdown data distributions, including the sex variable. All the analyses were conducted using the R software [33].

## 3. Results

**Figure 1:**
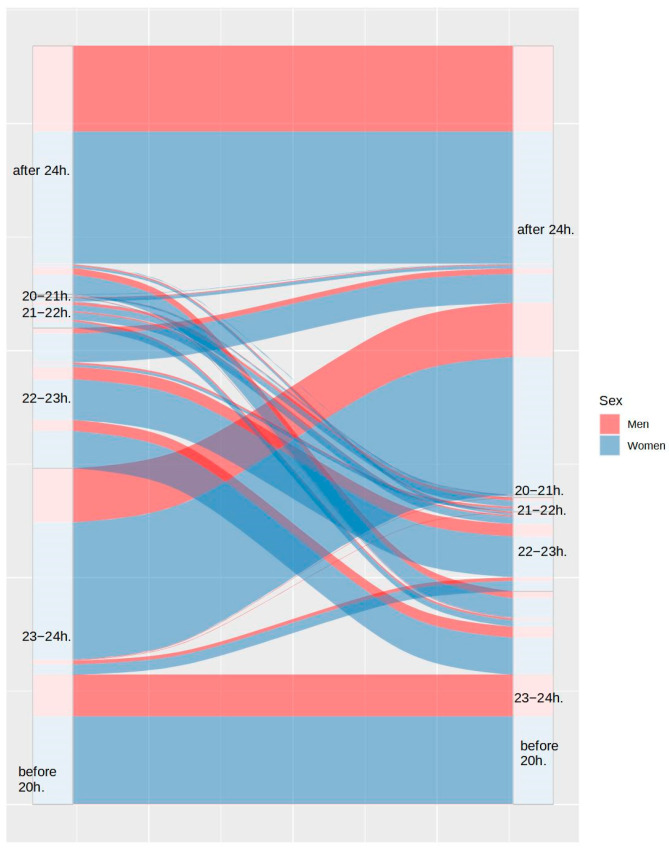
Differences in bedtime between the usual routine (left) and during lockdown (right), by sex.

**Figure 2:**
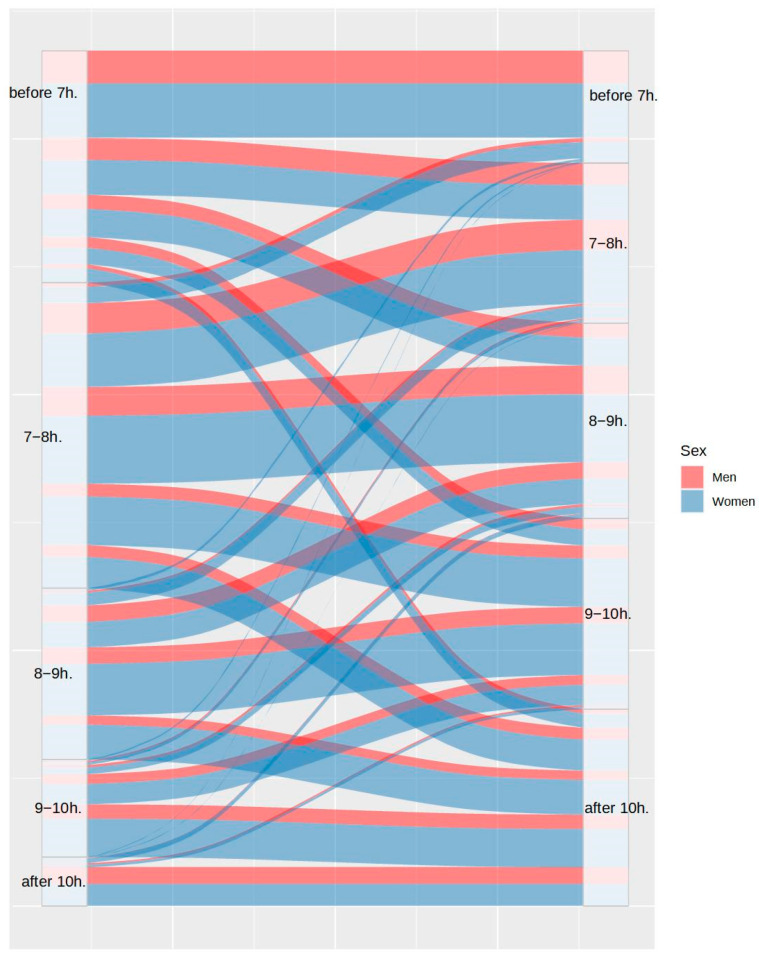
Differences in wake-up time between the usual routine (left) and during lockdown (right), by sex.

First, the proportion of participants with disturbances in their usual sleep patterns was analyzed. Figure 1 and Figure 2 show the differences between the bedtimes and the wake-up times, respectively. As can be observed in both figures, 54.23% of participants maintained their bedtimes, 39.14% of the remaining participants delayed their usual bedtime, and only 6.64% of participants advanced their bedtime. Despite these changes, only 32.95% of participants maintained their wake-up time, as 59.62% of them delayed it and 7.37% of them advanced it.

**Figure 3:**
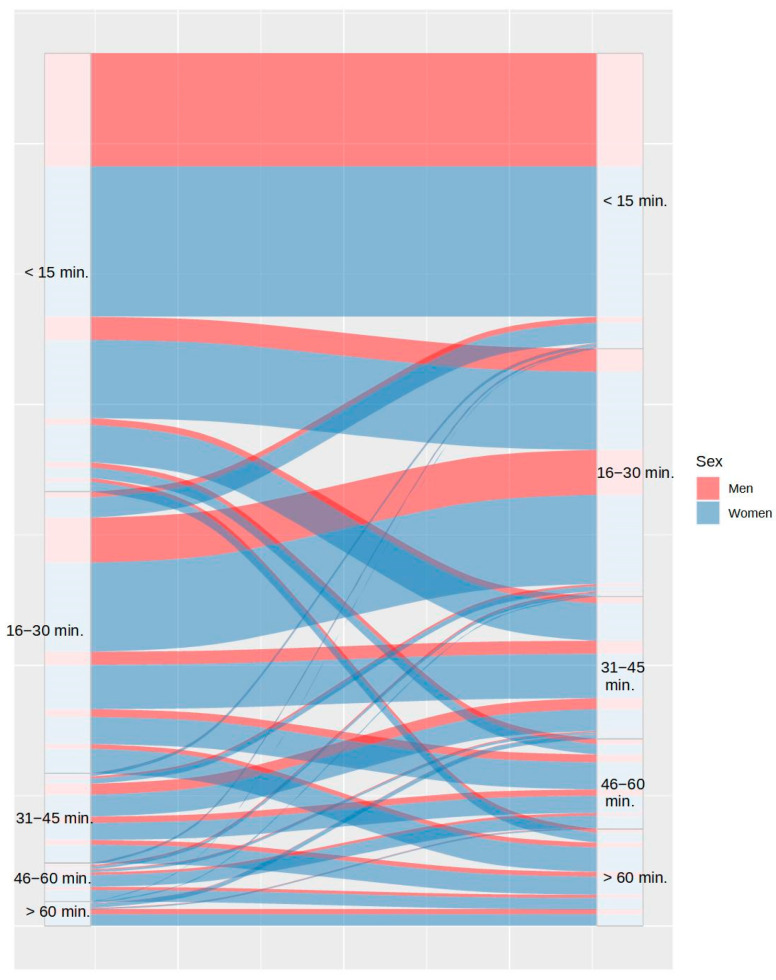
Differences in time to fall asleep between the usual routine (left) and during lockdown (right), by sex.

**Figure 4:**
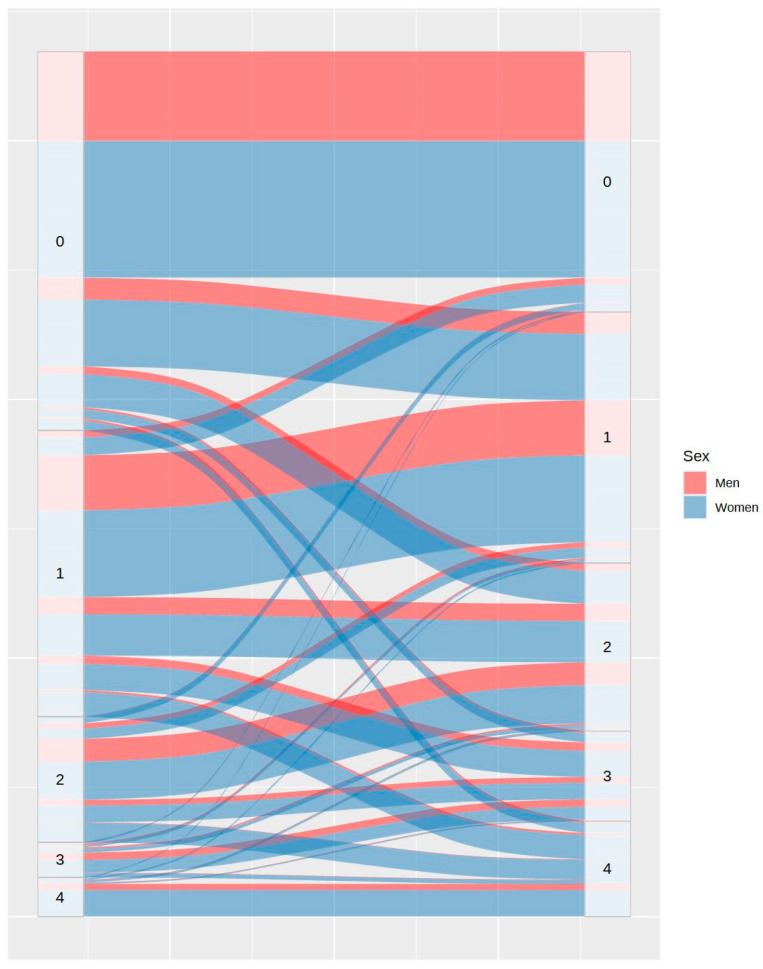
Differences in number of awakenings between the usual routine (left) and during lockdown (right), by sex.

**Figure 5:**
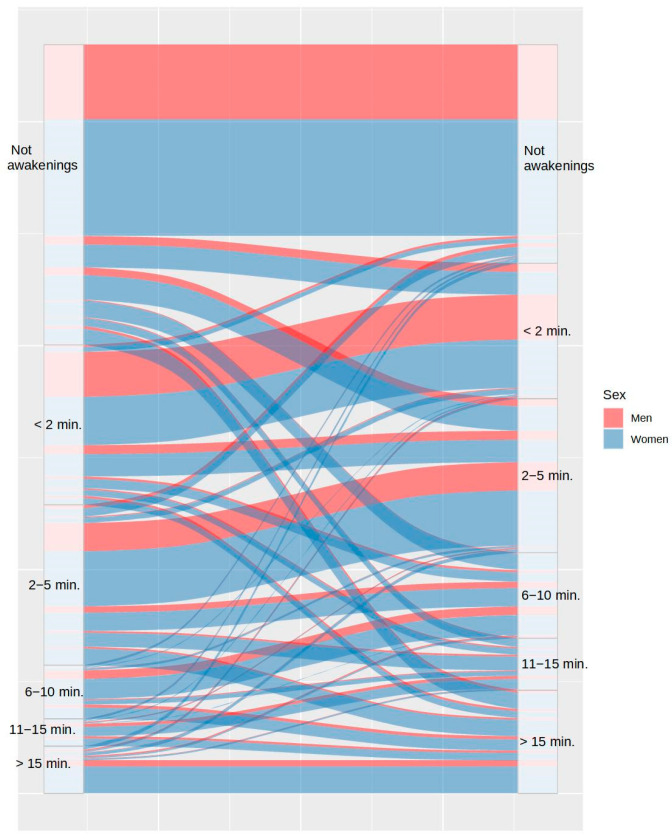
Differences in duration of awakenings between the usual routine (left) and during lockdown (right), by sex.

Concerning sleep onset latency, Figure 3 shows that 59.04% of participants took the same or lesser time to fall asleep, while 40.96% took more time to fall asleep during COVID-19 lockdown. Figure 4 shows the changes in the number of awakenings as a consequence of lockdown. As it can be observed, the number of awakenings increased in 36.91% of participants and decreased in only 7.43% of them, despite the greater freedom to modify sleep schedules. Regarding the mean time to fall asleep after night awakenings, Figure 5 shows that this only decreased in 3% of participants during lockdown and increased in 30.95% of them. It is remarkable that 14.54% of the participants who had never experienced night awakenings before did so during the lockdown.

**Figure 6:**
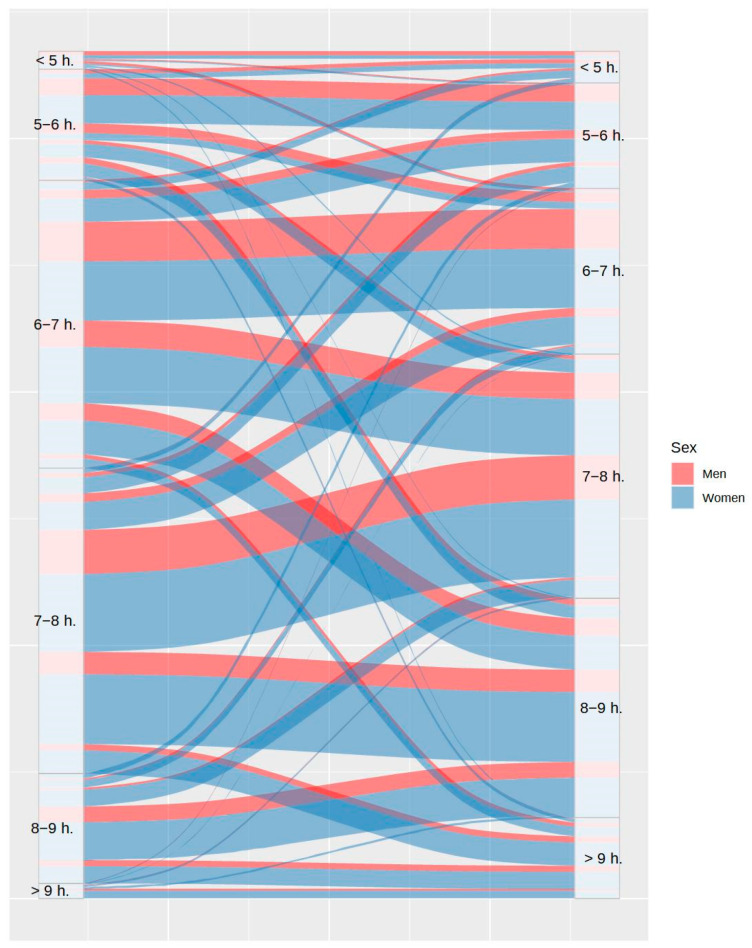
Variations in total sleep time between the usual routine (left) and during lockdown (right), by sex.

**Figure 7:**
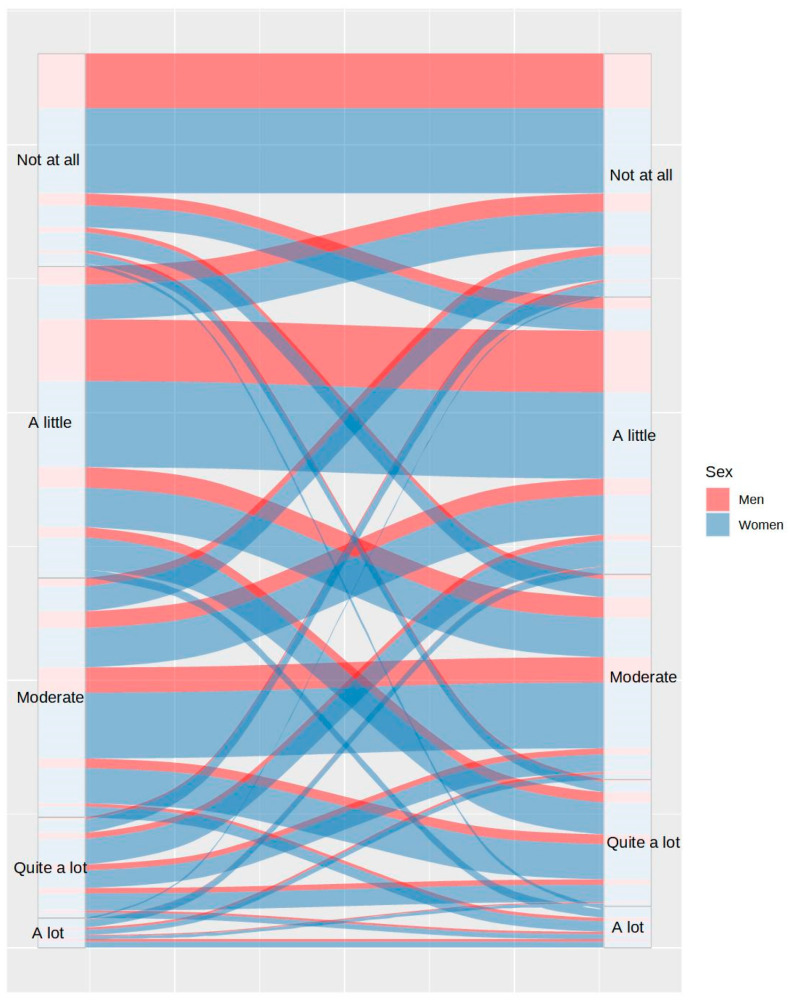
Differences in sleepiness between the usual routine (left) and during lockdown (right), by sex.

Beyond the variables related to sleep habits and schedules, information on other variables more directly related to sleep quality was also analyzed. It can be observed in Figure 6 that total sleep time was higher during lockdown in 42.42% of participants, while this decreased in 17.84% of them. Although people had more freedom to adjust sleep time during lockdown, and accordingly, they were supposed to sleep better, the proportion of participants with somnolence was almost equal prior and during lockdown (28% and 26.24%, respectively), as shown in Figure 7.

**Figure 8:**
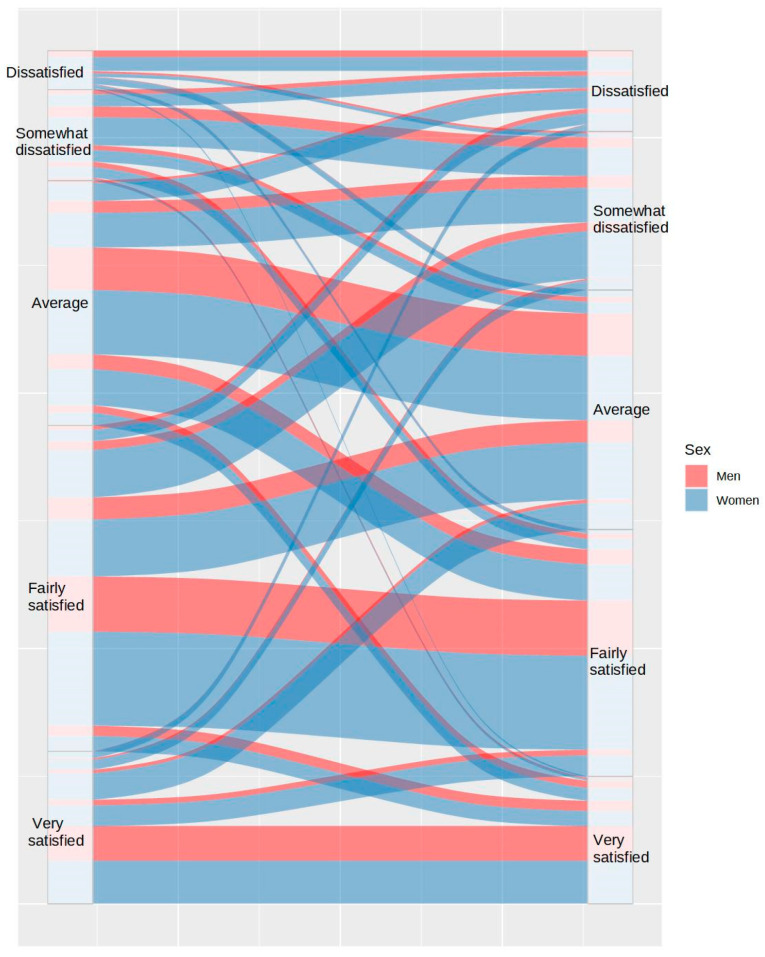
Differences in sleep satisfaction between the usual routine (left) and during lockdown (right), by sex.

**Figure 9:**
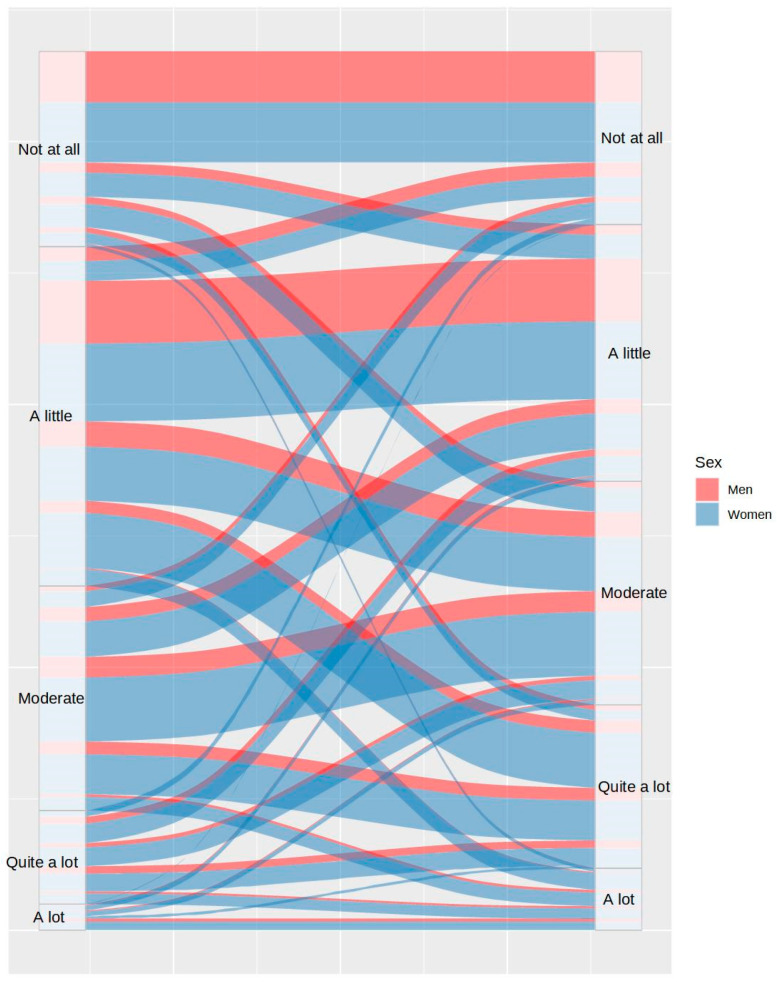
Differences in tiredness between the usual routine (left) and during lockdown (right), by sex.

Likewise, 36.37% of participants reported better sleep satisfaction prior to lockdown than during lockdown, while only 17.63% of participants reported better sleep satisfaction during lockdown (Figure 8). All of this influenced the fact that 37.6% of participants reported more daytime tiredness during lockdown, in contrast to 20% of participants, who reported lower tiredness (see Figure 9).

Finally, differences between both sexes in each of the variables analyzed and the pre- and during -lockdown comparison, according to the chi square tests, are shown in Table 1. As it can be observed, most of the distributions were different for men and women, with the latter manifesting more problematic sleep patterns both before and during lockdown. Moreover, it is also observed that sleep variables were significantly affected by lockdown.

## 4. Discussion

The main goal of this study was to analyze differences in sleep patterns after the declaration of the state of alarm. Data on bedtime, wake up time, sleep onset latency, the number and duration of awakenings, total sleep time, levels of daytime sleepiness, sleep satisfaction, and tiredness before and during lockdown were collected. The results obtained showed a noteworthy proportion of people with disturbances in their sleep schedules (45.66%). These results are consistent with those found by Cellini et al. [16] and Marelli et al. [17]. In this case, there were also 20% of participants who delayed their wake-up time, despite maintaining their same bedtime, entailing an increase in their number of total sleep hours during lockdown. This might be a sign of the usual sleep schedule-related problems when there is no lockdown. Moreover, although two out of three participants modified their sleep times, almost half of the participants took more time to fall asleep and experienced more and longer night awakenings. A high percentage of participants showed greater dissatisfaction with their sleep quality, daytime sleepiness, and fatigue.

Concerning sleep continuity measures, almost half of the participants experienced an increase in the number and duration of night awakenings. This supports previous results [34,35,36,37] and is consistent with the findings suggesting a worse sleep quality during lockdown. These results might also be related to the increase in daytime sleepiness and sleep dissatisfaction reported by a high percentage of participants.

The fact that sleep quality deteriorated during lockdown despite greater freedom to adjust sleep schedules could be attributed to the stress and anxiety experienced during the pandemic, as well as, in many cases, the fear of contracting a serious and deadly disease. The perception of stress or anxiety is considered one of the main obstacles to falling asleep [5,38], beyond other aspects related to diet and exercise [39,40,41]. In effect, the greater the perceived stress, the worse the sleep quality, and vice versa: the reduction in perceived stress is associated with an improvement of sleep quality [34]. In this case, both the change of habits and the fear of disease are two stressful events that can affect sleep quality.

In a study conducted in a Spanish population during lockdown, Sandín et al. [42] found that the most common fears corresponded to categories such as infection/disease/dead, social isolation, and work/income problems. It was proven that this context favored people to suffer an emotional impact manifested by worries, stress, hopelessness, depression, anxiety, nervousness, and restlessness. Similarly, they discovered that females were more vulnerable to these symptoms, constituting a risk factor and predictor factor for virus fear. These results are consistent with those of other studies in which men showed greater emotional stability [43]. Nevertheless, it cannot be ignored that the state of alarm has also affected other health-related problems and behaviors apart from psychological well-being [44]. In addition to this, the differences between both sexes are consistent with our results, as women showed greater sleep disturbances both before and during lockdown, and, furthermore, the worsening of sleep was marked in them in almost all the variables analyzed. These results also support those observed by Maestro-González et al. [25] and Paiva et al. [36]. Sex variations are also evident in other related studies [45]. For further information on objective sleep-related variables, see Sierra et al. [46].

This work presents several limitations. First, conducting an online recruitment can lead to participation bias, so responses may be different from those found in a face-to-face sample. The second limitation is that the sampling has resulted in an imbalance in sex and age, most likely because digital platforms—the media employed for sampling due to confinement—are used less frequently among older people. Even when the sample size helps to increase the generalizability of the results, the imbalance in age and sex makes it more difficult. Additionally, related to the sample, the lack of a control group does not allow to ensure that changes in sleep patterns were due to confinement and not affected by other uncontrolled factors. Third, not having standardized measures (decided in this way to maximize the response rate and minimize completion errors) can influence the reliability of the results. Despite this, the fact that the questions are fundamentally about objective facts helps to ensure adequate reliability. In effect, many of the most commonly used sleep measures (e.g., sleep diary) do not have results of reliability. Despite these limitations, the present work has made it possible to emphasize the importance of taking care of sleep in such stressful environments and when routines vary as much as during a lockdown.

## 5. Conclusions

The results of this study help to understand variations in sleep schedules and sleep quality during the COVID-19 lockdown. Sleep schedules varied by increasing the amount of time in bed in most cases. However, sleep quality got worse in a large proportion of the participants, according to their results in the rest of sleep variables assessed. The sources of these problems, despite having greater freedom to adjust schedules, need to be further studied.

## Figures and Tables

**Table 1:** Sex differences in sleep habits and sleep quality-related variables.

Variable	Sex		Total Sample	
	χ^2^ (DF)	*p*	χ^2^ (DF)	*p*
Bedtime pre-lockdown	29.4 (5)	<0.001	2902 (25)	<0.001
Bedtime lockdown	9.08 (5)	0.106		
Wake up time pre-lockdown	14.86 (4)	0.005	785 (16)	<0.001
Wake up time lockdown	6.74 (4)	0.15		
Time to fall asleep pre-lockdown	13.14 (4)	0.011	941 (16)	<0.001
Time to fall asleep lockdown	44.58 (4)	<0.001		
Awakening number pre-lockdown	7.76 (4)	0.1	1261 (16)	<0.001
Awakening number lockdown	44.42 (4)	<0.001		
Awakening duration pre-lockdown	21.5 (5)	<0.001	1810 (25)	<0.001
Awakening duration lockdown	60.68 (5)	<0.001		
Total sleep time pre-lockdown	18.34 (5)	0.003	721 (25)	<0.001
Total sleep time lockdown	13.14 (5)	0.022		
Sleepiness pre-lockdown	23.51 (4)	<0.001	569 (16)	<0.001
Sleepiness lockdown	19.1 (4)	<0.001		
Sleep satisfaction pre-lockdown	1.85 (4)	0.763	748 (20)	<0.001
Sleep satisfaction lockdown	31.86 (5)	<0.001		
Tiredness pre-lockdown	28.48 (4)	<0.001	596.42 (16)	<0.001
Tiredness lockdown	51.25 (4)	<0.001		

Note. DF = Degrees of freedom.

## Data Availability

Data available on request.
